# Monthly Report on the Sanitary Condition of Chicago and the Progress of Epidemics

**Published:** 1855-05

**Authors:** N. S. Davis


ART. II.—*Monthly Report on the Sanitary Condition of Chicago, and the Progress of Epidemics*, presented to the Cook Co. Medical Society, at its regular meeting, April 3rd, 1855. By N. S. DAVIS, M.D., &c. &c. &c.

The last Report from your Committee was made at the meeting of the Society, on the first Tuesday in February.

During the two months which have intervened (February and March), the weather has been unusually cold, with considerable snow, and the atmosphere has generally been dry. No disease has been sufficiently prevalent during these two months to merit the name of an epidemic; and yet there have been a few cases of disease worthy of mention.

---

*Dysentery*.—Ten cases of severe dysentery have come directly under my care during the past two months.

With only one exception, these were purely inflammatory in their nature and symptoms. The attacks were generally ushered in with chilliness, followed by active febrile action; characterized by a quick pulse, dry skin, deficient moisture in the mouth, frequent griping pains through the abdomen, especially in the course of the colon, with very frequent discharges of mucous and blood, small in quantity, but accompanied by protracted tenesmus.

If the disease continued for five or six days, the mucous discharged, which was at first glairy, became opaque, and generally mixed with pus, as well as blood; thereby showing a tendency to the early establishment of ulceration in the mucous membrane of the rectum and colon. None of the cases of dysentery or diarrhœa which have come under my observation during the last two months have presented those large serous evacuations, with cool skin, small pulse, and feeling of oppression at the epigastrium, which were often seen during the latter part of the winter and spring of 1854, and which preceded the severe prevalence of the cholera during the summer of that year.

Although watching closely, I have thus far seen nothing in connection with the intestinal diseases of the past two months, which indicate a tendency to cholera. This fact, in connection with the absence of all reports in regard to the existence of that much dreaded disease, at New Orleans and the Lower Mississippi, affords good reason to believe that we shall be compaartively exempt from it during the coming summer.

---

*Congestive Pneumonia.*—During the first part of March I met with more than the usual number of cases of pneumonia, and among them several which, in the commencement, presented symptoms strongly resembling those of pernicious fever. The following case will serve as a fair illustration :

March 9th, was called to Mr. P. D., a native of Ireland, aged 35 years, naturally strong and robust. Was attacked the evening previous rather suddenly, with *chilliness*, accompanied by a sense of weakness and distress throughout the whole system. The feeling of oppression in the chest and apparent prostration was was such as to alarm both the patient and his friends. At the time of my visit (8 o'clock A.M.), his face was of a dingy, or leaden color, with an expression of anxiety; the skin generally was cool, and the color under the nails purplish ; the pulse was 110 per minute, small, and easily compressed, but the impulse of the heart was more forcible; the tongue was covered with a whitish fur; the intestinal evacuations normal ; the breathing hurried and imperfect, with deep seated pain, or rather an indescribable sense of oppression and distress in the chest, mostly on the right side ; and some disposition to cough. Percussion elicited an undue degree of dulness over nearly the whole of the right side of the chest. Over the same side the respiratory murmur was diminished, the voice bronchial, and at the upper part of the left side the murmur was puerile.

I directed a large mustard sinapism to be applied over the epigastrium and right side, and when irritation was freely excited in the skin, to move the same to the dorsal spine. Also sinapisms to the extremities. Internally, I directed a powder, consisting of sulph. quinine 4 grs., pulv. opii 2 grs., and sub. murias hydrarg. 3 grs , every two hours until three doses were taken, and afterwards every four hours until the next visit.

March 10th, 9 o'clock A.M., I found the patient much relieved. He slept considerable during the afternoon and night previous, and sweated freely. His pulse was only 90 per minute, and more full; moderate heat of skin; some thirst; slight headache, and bowels quiet. But he had more cough, and a little blood, mixed with mucous expectoration; some difficulty of breathing, and sub-crepitunt rale over the central part of the right breast. I ordered castor oil sufficient to move the bowels, the operation to be followed by a powder consisting of sulph. quinine 3 grs., pulv. opii 1 gr., and tart. ant. et pot. 1-6 gr. given every four hours.

March 11th.—Symptoms still more improved; cough moderate; expectoration opaque, and free from blood; no pain in the chest, and only a moderate degree of mucous rhoncus.

Pulse 80 per minute and soft; skin moist and of natural temperature. Prescribed a simple expectorant mixture, with a single powder of quinine, pulv. opii. and pulv. bloodroot every night. In three days from the first visit, the patient was able to sit up, and in two more he left the house.

Between the 1st and 20th of March I was called to attend eight cases of this kind; and if they were treated early on essentially the plan just mentioned, they speedily recovered. But in every instance where the disease was not arrested during the first three days, the pulmonary tissue became extensively infiltrated; the expectoration being copious, thin, and very bloody; the chest decidedly dull on percussion; the respiration abdominal; the pulse soft and gaseous; the mind dull, and sometimes wandering. In a part of the cases there was a distinct remission in the symptoms every morning. In all the cases characterized by early and great depression, I found full doses of quinine opii, and calomel, with counter irritation, to afford most prompt relief Where the reaction became decided, with the skin dry and hot, the tart. ant. et pot. was added in such doses as the stomach would bear.

In the more advanced stages, when the expectoration was copious, pulv. bloodroot was given in the place of antimony. In a case which was brought to the hospital six days after the attack, with very copious and bloody expectoration, of a dark color, and liquid; leaden colored lips; abdominal respiration; marked dulness over the lower and central parts of both sides of the chest, with coarse mucous rhoncus and diffused broncophony; with much general prostration. I prescribed at once 15 drops of oil of turpentine, and 2 grs. of quinine, rubbed up with gum arabic and sugar, every four hours, and between each dose a powder, consisting of chloride of sodium 15 grs., sulph. quinine 2 grs., pulv. bloodroot, ½ gr., and opium 1 gr. Also a large blister to the chest.

Under this treatment, with an occasional laxative, the patient immediately began to improve, and in two weeks was quite convalescent. I have seen but one fatal case of this disease during the time embraced in this report; and in that death resulted on the fourth or fifth day, from overwhelming infiltration of the pulmonary tissue.

---

*Erysipelas.*—Since the first of February I have met with eight or ten cases of severe erysipelas. In all the cases but one, the disease was located on the face and head, and, though severe, presented nothing peculiar. In one case to which I was called after the patient had been sick two weeks or more, the disease commenced on the hip, and had spread until it covered both hips, the lower part of the back and gluteal regions, the thighs, scrotum and penis. All the parts were considerably swollen, especially the last named. There was also retention of urine, and much general prostration, with frequent, liquid, and almost involuntary discharges from the bowels. I evacuated the bladder by the catheter, and gave the patient an emulsion of oil of turpentine, tinct. of opii, and quinine, to control the bowels, and 20 drops of the muriated tincture of iron every four hours. He has steadily but slowly improved. The erysipelatous inflammation has entirely subsided, except a spot on one knee; the bowels have become regular; the appetite better; but the bladder still remains in a state of atony, some urine dribbling away involuntarily, and the rest removed by the catheter. In one of the cases of erysipelas of the face and head, I used the tinct. of iron without any benefit. The patient recovered under the use of quinine, and alterative doses of calomel, with cold applications to the inflamed surface. In another case the muriated tincture was partially successful, and in another promptly and fully so. In the remaining cases it was not used.

---

*Puerperal Fever.*—During the past two months I have been called to attend six cases of this much dreaded disease. None of them proved fatal, although two sunk to a very low condition. The treatment in all the cases was commenced early; and in all but one the principal reliance was on opium and calomel, in the proportion of 2 grs of the former to 3 grs. of the latter every two hours until sleep and freedom from pain was induced. When this effect was obtained the doses were diminished, and the interval between their administration lengthened, but the use of the remedies continued until the effects of the calomel on the gums could be detected. Then if the pulse remained quick and irritable, I prescribed the following, viz.:

Tinct. veratrum viride, - - ʒj.

Tinct. opii et camph. - - ℥ii.

Mix. Give one fluid drachm every four hours, with 20 drops of muriated tincture of iron between each dose. Anodyne fomentations were applied to the abdomen early and continuously as long as tenderness remained. In one case no opium or calomel was given, and it may be worth quoting at length.

Mrs. B.—Pregnant for the first time; had suffered moderately from gastric and intestinal irritation, manifested by repeated attacks of vomiting and diarrhœa during the greater part of the period of gestation. About four weeks before her confinement I had been callad upon to treat her for a decided attack of gastritis, so severe as to make her dangerously sick for several days. While attending her for this attack, I learned, by actual trial, that she would bear neither morphine nor any of the preparations of opium without the most protracted nausea and severe nervous prostration. Being called to attend her while two of the cases of puerperal fever just alluded to were under treatment, I took every precaution that I could without refusing to attend. And I should have done this, had it not been for the previous attendance on the patient, and the extreme disappointment she would feel from the refusal. Her labor was favorable, and no unfavorable symptoms occurred until the evening of the fourth day after delivery. She then complained of some uneasiness in the abdomen, and having had only one slight alvine evacuation since her confinement, the nurse gave her a laxative, followed by an enema of warm water, but it failed to procure any evacuation. In the night she was seized with rigors, and increased pain in the abdomen, and much restlessness. Early in the morning I was sent for, and found her with an anxious expression of countenance, hurried respiration, hot skin, abdomen moderately distended, and tender to the touch, especially over the lower part; lochial discharge very slight, and secretion of mi k diminished; pulse 180 per minute, and small, with a sense of distress in the abdomen, and much restlessness. Knowing the effects of opium upon her, I adopted the following treatment, viz.: applied fomentations of hops over the abdomen, and gave her the veratrum viride in combination as follows:

℞ Sat. tinct. veratrum viride, ʒi.
Spts. nit. dulc., - - ℥i.
Tinct. opii et camph., - ℥i.

Mix, and give one tea-spoonful every *two* hours. I watched the patient closely, and in twelve hours the pulse was reduced to 95 per minute; she was less restless, and the abdominal tenderness diminished. The treatment was continued; the drops being given at intervals of three hours, instead of two. The following morning, twenty-four hours after the commencement of the treatment, the pulse was only 85 per minute; the abdomen still bloated, but only slightly tender to pressure; the lochial discharge more free; the expression of countenance better; and the patient feeling comfortable, except slight nausea. I now ordered a large enema of warm water and common salt, which produced a free evacuation from the bowels, and lessened the fulness of the abdomen. Continued the veratrum mixture every four or six hours for two days, and the patient made a good recovery.

This is the second case of puerperal fever that I have treated with the veratrum viride as the chief internal remedy. The first case, I believe, was reported to this society on a former occasion.

Dr. Blaney inquired if any connection was observed between the cases of erysipelas mentioned in the report, and those of puerperal fever.

The reporter (Dr. Davis) replied that he was not satisfied that they had any connection with each other, either direct or indirect. Three of the cases of puerperal fever were not seen by him until the commencement of the fever, they having been attended in labor by *midwives*. They were not known to have any connection either with erysipelas or other cases of puerperal fever. The circumstances connected with one of the other cases he had just detailed.

Of the remaining two cases, one was in a patient to whom he was called after she had been in hard labor twenty-four hours under the care of a midwife. He found the patient still having frequent and severe labor pains, extremely restless, with frequent vomiting and a quick pulse. On examination, he found the hand and fore-arm of the child protruding from the labia, and the shoulder and scapula so firmly pressed down into the cavity of the pelvis as to nearly touch the perineum. The presenting parts of the child were much tumefied, and the vagina hot and dry. The condition of the woman indicating the necessity of as speedy a delivery as possible, he at once oiled the hand well, and after a steady, patient effort, succeeded in carrying it past the shoulder into the cavity of the womb, and by working carefully between the pains, finally succeeded in reaching the feet. By making traction on the feet with the hand in the womb, and at the same time pressing back the shoulder with the fore and middle fingers of the other hand, he turned the child, brought down the feet, and completed the delivery in little more than half an hour. The placenta was removed without difficulty, and the flowing was moderate. As the patient was much fatigued by the protracted labor, and the abdomen remained more distended and tympanitic than usual after delivery, he took the precaution to administer 1½ grs of opium with three of calomel, and directed it to be repeated every three hours until the patient got some sleep. She rested some during the night, and did not repeat the doses of medicine.

He found her the next day, however, with a rapid pulse, dry skin, distended and tender abdomen, and suppressed lochia. She was brought rapidly under the influence of opium and alteratives with fomentations to the abdomen, and finally recovered.

The sixth and last case occurred in the same house, and in immediate contact with a previous case of the same disease. The circumstances are as follows:

Two sisters lived in the same house. One of them had passed through the usual period of gestation, and had been delivered under the care of a midwife. About the fourth day after the delivery, she was taken with chills, and severe symptoms of puerperal peritonitis, and the Doctor was called in. While attending this patient, the sister, who was about seven months advanced in pregnancy, was taken with labor pains, and soon expelled a dead and diseased fœtus. While attending her in labor, he found the labia ragged with chancres, and buboes in the groin. He soon found that both the women were affected with primary syphilis. Symptoms of peritonitis were subsequently developed in the second patient as severe as in the first.

Dr. Blaney remarked that he was fully satisfied that erysipelas was capable of communicating to lying-in women puerperal peritonitis; and also that the latter disease is highly contagious. Of the latter fact he had been so well satisfied, that for several years he had refused to attend cases of labor whenever he was in direct attendance on either of the diseases named.

Dr. Bloodgood stated that he had known one instance where a physician, while attending daily a bad case of erysipelas, in which suppuration had taken place, attended also *nine* cases of labor. And in every instance the labor was followed in a few days by puerperal peritonitis, which as uniformly terminated fatally.

